# Association of Neutrophil to Lymphocyte Ratio With Plaque Rupture in Acute Coronary Syndrome Patients With Only Intermediate Coronary Artery Lesions Assessed by Optical Coherence Tomography

**DOI:** 10.3389/fcvm.2022.770760

**Published:** 2022-03-10

**Authors:** Jintong Jiang, Huasu Zeng, Yang Zhuo, Changqian Wang, Jun Gu, Junfeng Zhang, Huili Zhang

**Affiliations:** Department of Cardiology, Shanghai Ninth People’s Hospital, Shanghai Jiao Tong University School of Medicine, Shanghai, China

**Keywords:** intermediate coronary artery lesion, plaque rupture, optical coherence tomography, neutrophil to lymphocyte ratio (NLR), atherosclerosis

## Abstract

**Objectives:**

Plaque vulnerability and rupture rather than plaque size are the major cause of clinical events in patients with intermediate coronary lesions. Therefore, the present study was aimed to explore potential markers associated with plaque rupture in acute coronary syndrome (ACS) patients with intermediate coronary lesions.

**Methods:**

A total of 82 ACS patients presenting with only intermediate coronary lesions (40–70% stenosis demonstrated by quantitative coronary angiography) and no severe stenosis in other main coronary arteries [median age 63 years, 53 male and 29 female] were enrolled. Plaque morphology were assessed by optical coherence tomography (OCT). Hematological indices were assayed by automated hematological analyzer.

**Results:**

Plaque rupture was identified in 14 patients by OCT. Neutrophil to lymphocyte ratio (NLR) in patients with plaque rupture (*n* = 14) was significantly higher than that in patients with non-plaque rupture (*n* = 68) [3.85 (3.28, 4.77) vs. 2.13 (1.40, 2.81), *p* < 0.001]. Multivariate logistic regression analysis revealed that NLR was one of the independent risk factors for plaque rupture in intermediate coronary artery lesions (odds ratio 1.64, 95% confidence intervals 1.18–2.29, *p* = 0.003). ROC curve analysis found a cutoff point of NLR > 2.94 for plaque rupture with 93.8% sensitivity and 77.9% specificity.

**Conclusion:**

NLR, an inflammatory biomarker, is closely associated with plaque rupture in intermediate coronary artery lesions. Monitoring NLR may be useful in risk stratification and management for intermediate coronary artery lesions.

## Introduction

Intermediate coronary artery stenosis is defined as an angiographic stenosis of 40–70% (1, 2). It may account for up to 25% of patients undergoing coronary angiography (3). Optimal assessment and interventional strategy for intermediate lesions continues to be a clinical challenge for cardiologists. Therefore, it is meaningful to explore potential biomarkers which can provide useful information on pathophysiology, risk stratification and management for this condition.

Numerous studies have shown that plaque vulnerability and rupture rather than plaque size and stenosis severity are the major cause of cardiovascular events in patients with coronary artery disease (CAD) (4, 5). The process of plaque rupture may be attributed to neutrophil infiltration and subsequent neutrophil-platelet adhesion. Neutrophil infiltration in disrupted plaques and tissue damage caused by neutrophil elastase were directly visualized by immunohistochemistry in the arteries of the circle of Willis from human autopsy cases (6). Animal studies suggest that infiltrated neutrophils make plaques prone to rupture by releasing proteolytic enzymes, superoxide radicals and inflammatory mediators (7, 8).

Neutrophil to lymphocyte ratio (NLR), reflecting the combination of neutrophil and lymphocyte alterations is currently proposed as an accurate biomarker with predictive power for cardiovascular adverse events in patients with acute coronary syndrome (ACS), stable CAD patients and patients undergoing coronary artery bypass graft (CABG) (9–13). Unfortunately, to the best of our knowledge, there is a paucity of the association of NLR with plaque vulnerability in CAD patients, especially in patients with intermediate lesions. Given this background, the present study was aimed to investigate the possible link between NLR and plaque rupture in patients with only intermediate coronary lesions in the acute phase of ACS.

Optical coherence tomography (OCT) is a catheter-based imaging modality that provides detailed visualization of intraluminal coronary artery structures. By OCT imaging, cardiologists are able to observe the plaque morphology and composition *in vivo*, such as thin-cap fibroatheroma, plaque erosion, plaque rupture and calcified nodule (14, 15). Therefore, we used OCT to identify plaque rupture in ACS patients with only intermediate coronary lesions and then compared blood cell counts, hematological indices and inflammatory response between patients with and without plaque rupture.

## Materials and Methods

### Study Population

This is a prospective observational study conducted at Shanghai Ninth People’s Hospital, Shanghai Jiaotong University School of Medicine. Our study complied with the declaration of Helsinki and was approved by the hospital ethnics review board (No. 2016-256-T191). Informed consent was obtained from all the participants. A total number of 186 consecutive ACS patients were evaluated for enrollment in this study during the period from September 2016 to December 2020. All the patients received elective coronary angiography and OCT within 72 hours after admission. A total number of 82 ACS patients with only intermediate coronary lesions were finally enrolled and evaluated. One hundred and four ACS patients were excluded from the study because they also had severe stenosis (>70%) in other main coronary arteries. ACS was clinically diagnosed according to the elevated cardiac biomarkers, abnormal electrocardiogram indicating myocardial ischemia, clinical symptoms of angina pectoris or cardiac wall motion abnormalities by echocardiography. ACS included ST-segment elevation myocardial infarction (STEMI), non-ST-segment elevation myocardial infarction (NSTEMI), and unstable angina pectoris (UA). Patients with severe valvular heart disease, severe heart failure, infectious disease, autoimmune diseases, serious lung diseases, sever liver and kidney disease, malignant tumor, hemorrhage, and hematologic disorders were excluded from the study.

All patients were routinely treated with anti-platelet drugs, statins, β-blocker or angiotensin converting enzyme inhibitor or angiotensin II receptor blocker. Demographic information, clinical data and laboratory findings were collected. All the patients were followed up every 3 months for a median of 35.0 months [18.0, 45.5]. Major adverse cardiac events (MACE) were recorded during the period of follow-up. MACE was defined as the composite of all cause death, cardiac death, MI, coronary revascularization and stroke.

### Coronary Angiography

All participants underwent elective coronary angiography by the standard Judkin’s technique. Coronary angiograms were assessed and quantified separately by two independent interventional physicians blind to the clinical data. An intermediate coronary lesion was defined as a luminal narrowing with a diameter stenosis of 40–70% in any of the main coronary arteries, including the left main artery (LM), left anterior descending artery (LAD), left circumflex coronary artery (LCX), and right coronary artery (RCA), or main branches of the vascular systems (the diameter of the target vessel is ≥ 2.5 mm).

### Optical Coherence Tomography Procedure and Imaging Analysis

**FIGURE 1:**
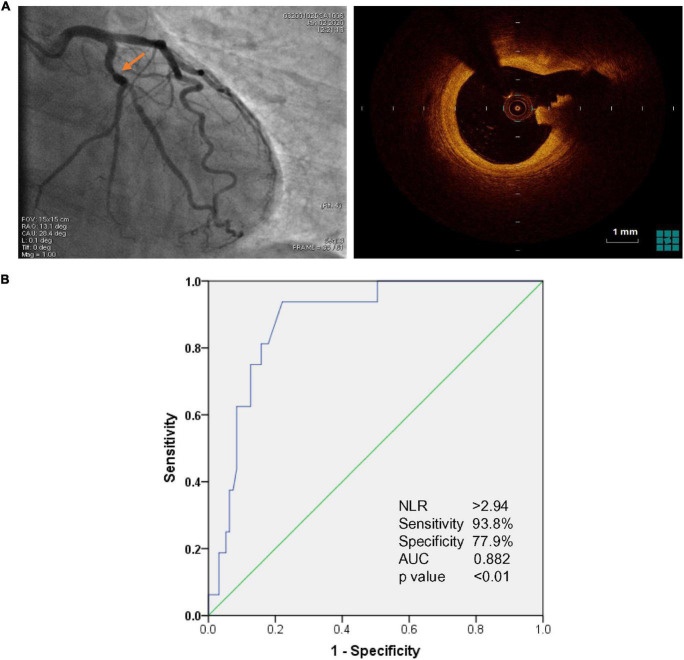
**(A)** Representative image of optical coherence tomography (OCT)-identified plaque rupture (right panel) in angiographically intermediate coronary lesions (arrowhead in left panel). **(B)** Receiver Operating Characteristic (ROC) for evaluation of Neutrophil to lymphocyte ratio (NLR) associated with plaque rupture in acute coronary syndrome (ACS) patients with intermediate coronary artery lesions. The cutoff value of NLR associated with plaque rupture was NLR > 2.94 with 93.8% sensitivity and 77.9% specificity [Area Under Curve (AUC): 0.882, CI 95% 0.809–0.955, *p* < 0.01].

All the intermediate lesions were assessed by OCT. OCT images were acquired using ILUMIEN OPTIS System with Dragonfly OPTIS imaging Catheter (Abbott Vascular, Santa Clara, CA, United States) and analyzed by two independent investigators who were blinded to the clinical presentations and angiographic findings. Morphological evaluation of intermediate lesions included plaque composition, plaque rupture, erosion, calcified nodule, vasospasm, and thrombus (14, 15). Plaque rupture was defined as the presence of a fibrous cap discontinuity and cavity formation in the plaque (Figure 1A).

### Blood Examination

Blood samples for evaluating hematological parameters and biomarkers of cardiac injury and inflammation were collected from a large antecubital vein of each patient immediately after admission. Blood samples for assaying biochemical parameters were collected after a 12-h overnight fast usually within 24 hours after admission. Blood cell counts and hematological indices were determined using an automated blood cell counter, the Coulter LH780 Hematology Analyzer (Beckman Coulter Ireland Inc.). Serum levels of total cholesterol (TC), low-density lipoprotein cholesterol (LDL-C) and high-density lipoprotein cholesterol (HDL-C), and triglycerides (TG) were measured by biochemical auto-analyzer (Siemens Advia 2400). NLR was calculated by dividing neutrophil count to lymphocyte count. Platelet to lymphocyte ratio (PLR) was calculated by dividing platelet count to lymphocyte count.

### Statistical Analysis

Statistical analysis was performed with SPSS version 21.0. Continuous variables were presented as median [first quartile, third quartile] and compared using the Mann-Whitney *U* test. Categorical variables were expressed as percentage and compared using the chi-square-test. Clinical features and hematological indices with *p*-value < 0.10 in univariate analysis were included in multivariate logistic regression analysis. Multivariate logistic regression analysis with forward stepwise was performed to evaluate the independent factors associated with the risk of plaque rupture. The odds ratios (OR) and 95% confidence intervals (CI) were calculated. Areas under the receiver-operator characteristic (ROC) curve for NLR related to plaque rupture were conducted. A two-tailed *p*-value of < 0.05 was considered statistically significant.

## Results

A total of 82 ACS patients (median age 63 years [57, 70], 53 male and 29 female) with only intermediate coronary lesion were enrolled and investigated. Sixty-six patients were diagnosed with UA. Fourteen and two patients were diagnosed with NSTEMI and STEMI, respectively. A total of 124 lesions (2 lesions in LM, 69 lesions in LAD, 18 lesions in LCX, and 35 lesions in RCA) were assessed by OCT. The OCT findings were described as follow: 35.7% (45/124) fibrous plaques, 22.6% (28/124) fibroatheroma, 8.1% (10/124) fibrocalcific plaques, 16.9% (21/124) mixed plaque, 11.3% (14/124) plaque rupture, 1.6% (2/124) spasm, 1.6% (2/124) dissection, 0.81% (1/124) intimal ulceration/erosion, and 0.81% (1/124) calcified nodule.

Patients were divided into rupture group (*n* = 14) and non-rupture group (*n* = 68) based on the OCT findings. Their demographic and clinical characteristics as well as laboratory data were summarized in Table 1. As shown in Table 1, patients in rupture group were significantly younger than non-rupture group. They also had higher proportions of male gender and current smokers as well as an increased level of Troponin I in comparison with non-rupture group. No substantial difference was observed between the two groups in terms of BNP, CRP, lipid profiles, hypertension, diabetes, and CAD family history. After follow-up for a median of 35 months [18.0, 45.5], an obvious difference was observed in the incidence of MACE between rupture and non-rupture group (3/14 vs. 3/68, *p* = 0.026). Three patients (1 NSTEMI and 2 recurrent UA) in non-rupture group underwent revascularization because of exacerbation of stenosis severity in intermediate coronary lesions (>70% stenosis). In rupture group, one recurrent NSTEMI and one recurrent UA occurred. These two patients received drug-eluted stent implantation as the diameter stenosis caused by intermediate lesions dramatically increased to > 70%. One patient in rupture group got ischemic stroke at 2-year follow-up.

**TABLE 1:** Baseline demographic and clinical characteristics of patients with or without plaque rupture.

	Plaque rupture (*n* = 14)	Non-plaque rupture (*n* = 68)	*p*-value
Age (years)	57 [51, 65]	64 [60, 70]	0.005
Male	12 (85.7%)	39 (57.4%)	0.046
Current smoking	10 (71.2%)	18 (26.5%)	0.001
Hypertension	9 (64.3%)	42 (61.7%)	0.859
Diabetes mellitus	2 (14.3%)	11 (16.2%)	0.860
Family history of CAD	3 (21.4%)	10 (14.7%)	0.531
Aspirin and/or thienopyridine	12 (85.7%)	60 (88.2%)	0.793
Beta blocker	4 (28.6%)	16 (23.5%)	0.689
ACEI/ARB	6 (42.9%)	26 (38.2%)	0.859
Statin	8 (57.1%)	35 (51.5%)	0.699
BMI (kg/m^2^)	23.49 [21.34, 27.09]	24.56 [23.25, 26.65]	0.346
TG (mmol/L)	1.63 [0.97, 2.12]	1.63 [1.18, 2.15]	0.810
TC (mmol/L)	3.91 [3.47, 4.28]	4.01 [3.52, 4.81]	0.820
LDL-C (mmol/L)	2.73 [2.18, 3.21]	2.70 [2.12, 3.47]	0.844
HDL-C (mmol/L)	0.99 [0.84, 1.13]	1.01 [0.90, 1.18]	0.824
Troponin I (ng/ml)	0.07 [0.00, 0.71]	0.01 [0.00, 0.03]	0.003
BNP (pg/ml)	50.00 [25.75, 109.25]	55.00 [25.00, 106.00]	0.900
CRP (mg/L)	1.99 [1.28, 3.89]	1.28 [1.28, 3.45]	0.074
eGFR (ml/min)	79.54 [52.08, 99.28]	64.30 [50.68, 78.40]	0.068

*Values are expressed as n (%) or median [first quartile, third quartile]. CAD, coronary artery disease; ACEI, angiotensin converting enzyme inhibitor; ARB, angiotensin II receptor blocker; TC, total cholesterol; TG, triglyceride; LDL-C, low density lipoprotein cholesterol; HDL-C, high density lipoprotein cholesterol.*

Table 2 showed the comparison of hematological parameters between rupture and non-rupture groups. Total number of WBC and differential count of neutrophils in rupture group were significantly higher than those in non-rupture group (both *p* < 0.01) whereas the lymphocyte count in rupture group was much lower than non-rupture group (*p* < 0.01). Median NLR was 3.85 [3.28, 4.77] and 2.13 [1.40, 2.81] in rupture and non-rupture group, respectively. NLR in rupture group was significantly higher than that in non-rupture group (*p* < 0.001). Median PLR was 169.45 [127.81, 210.21] and 121.62 [92.72, 160.04] in rupture and non-rupture groups, respectively and the difference between two groups was statistically significant (*p* = 0.002). Additionally, the two groups had similar levels of red blood cell count, hemoglobin, mean corpuscular hemoglobin (MCH), mean corpuscular volume (MCV), red blood cell distribution width (RDW), monocyte count, platelet count, mean platelet volume (MPV), and platelet distribution width (PDW).

**TABLE 2:** Hematological indices of patients with or without plaque rupture.

	Plaque rupture (*n* = 14)	Non-plaque rupture (*n* = 68)	*p*-value
Red blood cells (×10^12^)	4.54 [4.26, 4.80]	4.33 [3.99, 4.68]	0.530
Hemoglobin (g/L)	140.50 [128.00, 146.75]	134.00 [124.00, 141.00]	0.345
MCH (pg)	30.40 [29.70, 31.58]	30.30 [29.22, 31.40]	0.426
MCV (fL)	89.85 [88.53, 93.35]	90.10 [87.40, 92.70]	0.786
RDW (%)	13.10 [12.80, 13.50]	13.00 [12.40, 13.50]	0.286
White blood cells (×10^9^)	7.50 [6.73, 8.15]	6.10 [5.10, 6.90]	0.006
Neutrophils (×10^9^)	4.90 [4.00, 5.90]	3.50 [2.60, 4.40]	0.001
Lymphocytes (×10^9^)	1.30 [1.10, 1.50]	1.70 [1.30, 2.00]	0.002
Monocytes (×10^9^)	0.45 [0.39, 0.80]	0.44 [0.34, 0.60]	0.232
NLR	3.85 [3.28, 4.77]	2.13 [1.40, 2.81]	0.000
Platelets (×10^9^)	228.00 [143.75, 262.00]	206.00 [178.50, 224.75]	0.080
MPV (fL)	10.05 [9.93, 10.88]	10.40 [9.90, 10.80]	0.887
PDW (%)	11.85 [10.60, 13.18]	12.30 [11.20, 13.20]	0.707
PLR	169.45 [127.81, 210.21]	121.62 [92.72, 160.04]	0.002

*Values are expressed as n (%) or median [first quartile, third quartile]. MCH, Mean corpuscular hemoglobin; MCV, Mean corpuscular volume; MPV, Mean platelet volume; NLR, Neutrophil to lymphocyte ratio; PLR, Platelet to lymphocyte ratio; PDW, Plate distribution width; RDW, Red blood cell distribution width.*

Logistic regression analysis was performed to identify potential risk factors of plaque rupture in ACS patients with only intermediate coronary lesions (Table 3). Multivariate logistic regression analysis showed that NLR (OR1.64, 95% CI 1.18–2.29, *p* = 0.003) and status of current smoking (OR 6.74, 95% CI 1.61–28.21, *p* = 0.009) were independent factors associated with the risk of plaque rupture in intermediate lesions. Moreover, ROC curve analysis identified that NLR of 2.94 was an optimal cutoff value associated with the risk of plaque rupture in ACS patients with only intermediate lesions. NLR > 2.94 had 93.8% sensitivity and 77.9% specificity (AUC 0.882, CI 95% 0.809–0.955, *p* < 0.01) for predicting plaque rupture (Figure 1B).

**TABLE 3:** Logistic regression analysis for plaque rupture in patients with intermediate coronary artery lesions.

	Univariate			Multivariate		
	OR	95%CI	*p*-value	OR	95%CI	*p*-value
Age < 63 years*	3.62	0.96–11.80	0.058			
Male	4.46	0.93–21.49	0.062			
Current smoking	6.94	1.93–24.94	0.001	6.74	1.61–28.21	0.009
Troponin I	1.95	0.88–4.33	0.101			
WBC	1.41	1.03–1.94	0.031			
Neutrophils	1.65	1.14–2.37	0.007			
Lymphocytes	0.10	0.02–0.50	0.005			
NLR	1.73	1.21–2.46	0.002	1.64	1.18–2.29	0.003
PLR	1.02	1.00–1.03	0.002			

*Age was categorized according to the median of age distribution (<63 years or ≥63 years).

In addition, sex differences in inflammatory markers were analyzed. As shown in Supplementary Table 1, male patients in rupture group had more WBC and neutrophils, less lymphocytes as well as higher NLR and PLR in comparison with male patients in non-rupture group. In contrast, female patients in non-rupture and rupture group had similar levels of WBC, leukocyte differential count, NLR and PLR. No sex difference in inflammatory markers was obtained within rupture or non-rupture group.

## Discussion

Plaque rupture has been shown to be associated with the risk of MACE in patients with angiographically intermediate coronary artery lesions (16, 17). Exploring potential biomarkers associated with plaque rupture will be helpful in the follow-up of intermediate lesions and risk monitoring. NLR is a well-established and easily accessible inflammatory marker. Its prognostic value in CVD and predictive power for MACE have been highlighted by numerous epidemiological studies (9–13). Therefore, the present study assessed the plaque features in intermediate lesions by OCT and then explored the association of NLR with plaque rupture. We found that inflammation is more active in plaque rupture and elevated NLR is one of the independent risk factors for plaque rupture in intermediate lesions in the acute phase of ACS. The optimal cut-off point of NLR for plaque rupture in ACS patients with only intermediate lesions is >2.94 with 93.8% sensitivity and 77.9% specificity. Our study suggests that NLR, an inexpensive and readily available marker may provide useful information on cardiovascular risk assessment and management in intermediate coronary lesions. To the best of our knowledge, it is first study to investigate the relationship between NLR and acute atherosclerotic events, for instance plaque rupture in intermediate lesions.

Since chronic inflammation in arterial walls plays an essential role in every stage of atherosclerosis, the present study investigated the profiles of circulating leukocytes in patients with only intermediate lesion. We found an obvious elevation in neutrophil count and a pronounced decrease in lymphocyte count in plaque rupture group although their total WBC count was still within the normal range. As a results, NLR significantly raised in plaque rupture group. The close correlation between increased neutrophils and acute cardiovascular events is well-established because the presence of neutrophil infiltration has been identified at the site of plaque rupture in both animal models and human autopsy cases (6–8). Moreover, lymphopenia is another common hematological finding observed in ACS or MI (18–20). Lymphopenia has recently been suggested to be associated with increased risk of long-term mortality in patients undergoing CAG, regardless of the coronary presentation (21). Several possible mechanisms may account for the reduction of circulating lymphocytes in acute cardiovascular events (22, 23). During the acute setting of coronary artery disease, activation of immune system increased lymphocyte apoptosis in atherosclerotic lesions, resulting in the destabilization of atherosclerotic plaques (22). In response to physiological stress, a release of cortisol, catecholamines, and proinflammatory cytokines may lead to lymphopenia (23). Acute stress also caused the redistribution of lymphocytes to lymphoid organs and the inversion of CD4^+^/CD8^+^ T lymphocyte ratio (23).

Of note, NLR is a better marker of inflammation associated with plaque rupture than a single white blood cell count. Univariate logistic regression showed that neutrophil and lymphocyte differential counts as well as NLR were associated with plaque rupture. Nevertheless, by multiple logistic regression analysis, only NLR was identified as one of the independent factors associated with plaque rupture. Thus, NLR is likely to be a more accurate and reliable biomarker for evaluating inflammatory status and the risk of plaque rupture in intermediate lesions, as it reflects the combination of neutrophil and lymphocyte alterations in atherosclerotic events. In another word, ACS patients with only intermediate lesions should be cautiously monitored and followed-up when they have increased NLR > 2.94 (NLR cut-off value of 2.94 was observed in our study). Unfortunately, we did not analyze the predictive value of NLR to the long-term outcome of intermediate lesions in ACS patients because of the limited number of MACE observed during the follow-up. It warrants further investigation to elucidate whether NLR is a prognostic factor in patients with intermediate lesions.

As platelets contribute to the pathophysiology of ACS, the present study assessed the alterations of platelet indices (PLR, PDW, and MPV) in intermediate lesions. PLR dramatically increased in plaque rupture group while PDW and MPV remained unchanged. Unfortunately, PLR was not closely related to plaque rupture in intermediate lesions analyzed by multivariate analysis although it has been suggested to have a prognostic value in ACS patients (24, 25). On the other hand, it is worth emphasizing that EDTA, an anticoagulant used in blood sample collection may induce platelet swelling and thus increasing the value of PDW and MPV (26). Some medications, such as statin also have effect on the variability in platelet size in CAD (27). Thus, the link between platelet indices and plaque rupture still warrants further investigation and these factors affecting platelet features should be taken into account in our future study. Additionally, other hematological parameters, such as monocyte count and RDW did not alter in plaque rupture group. Taken together, our findings show that platelet indices and RDW are not powerful indicators for plaque rupture in intermediate lesions.

In addition to NLR, we found that the status of current smoking is another attributor to the plaque rupture in intermediate lesions [OR 6.74, 95%CI 1.61–28.21]. It has been well-documented that cigarette smoke destabilizes plaques and promotes plaque rupture by enhancing inflammation, activating matrix metalloproteinases, stimulating platelet activation and shifting the balance of hemostasis toward thrombus formation (28, 29). Smoke quitting causes an exponential reduction in acute cardiovascular events, particularly in the first year after cessation (30). Therefore, smoke cessation is highly recommended in ACS patients although they have only intermediate coronary lesions, especially those whose NLR is more than 2.94.

### Limitations of the Study

We recognized that there were several limitations in our study. Firstly, this prospective and observational study was conducted in a single center with a relatively small sample size. NLR was assayed in ACS patients with only intermediate lesions at admission. It is difficult to discriminate that NLR is upregulated before or after the event of plaque rupture. Thus, it is insufficient to provide a causative explanation for the association between NLR and plaque rupture in intermediate lesions. Secondly, it is difficult to analyze the prognostic value of NLR in intermediate coronary lesions because of the small sample size and the limited number of MACE. Thirdly, our findings may not reflect the alterations in female patients since the majority of the study population was male, especially in plaque rupture group. Large prospective cohort studies are required to obtain a better understanding between NLR and plaque rupture in intermediate lesions.

## Conclusion

Increased NLR is associated with OCT identified-plaque rupture in ACS patients with only intermediate coronary artery lesions. NLR may be used as a potential biomarker for plaque rupture in risk stratification and management for intermediate coronary artery lesions.

## Data Availability Statement

The raw data supporting the conclusions of this article will be made available by the authors, without undue reservation.

## Ethics Statement

The studies involving human participants were reviewed and approved by the Ethnics Review Board of Shanghai Ninth People’s Hospital, Shanghai Jiaotong University School of Medicine. The patients/participants provided their written informed consent to participate in this study.

## Author Contributions

JJ and HZe performed data collection and analysis and wrote the manuscript. YZ and CW performed CAG and OCT. JG contributed to data interpretation and assisted in conducting the study. HZh and JZ were the principal investigators and provided study design, interpretation, manuscript drafting and editing. All authors contributed to the article and approved the submitted version.

## Conflict of Interest

The authors declare that the research was conducted in the absence of any commercial or financial relationships that could be construed as a potential conflict of interest.

## Publisher’s Note

All claims expressed in this article are solely those of the authors and do not necessarily represent those of their affiliated organizations, or those of the publisher, the editors and the reviewers. Any product that may be evaluated in this article, or claim that may be made by its manufacturer, is not guaranteed or endorsed by the publisher.
