# Supplementary material for: Association of Neutrophil to Lymphocyte Ratio With Plaque Rupture in Acute Coronary Syndrome Patients With Only Intermediate Coronary Artery Lesions Assessed by Optical Coherence Tomography
Source: Front Cardiovasc Med. 2022 Mar 10;9:770760. doi: 10.3389/fcvm.2022.770760 (PMC8960066; doi:10.3389/fcvm.2022.770760)
Supplement: Supplementary file 1 [file Table_1.DOCX]

Table S1 Inflammatory markers in male and female ACS patients with intermediate lesions

|  | Male (n=53) | | |  | Female (==29) | | | *p_3_* value | *p_4_* value | *p_5_* value |
| --- | --- | --- | --- | --- | --- | --- | --- | --- | --- | --- |
|  | Non-rupture (n=41) | Rupture (n=12) | *p_1_* value |  | Non-rupture (n=27) | Rupture (n=2) | *p_2_* value |  |  |  |
| WBC (×10^9^) | 5.50[5.10, 6.65] | 7.60 [7.25, 8.38] | 0.003 |  | 6.10[4.90, 7.40] | 6.80, 5.30 | 0.897 | 0.665 | 0.132 | 0.677 |
| Neutrophil (×10^9^) | 3.50[2.60, 4.20] | 5.30[4.65, 6.23] | 0.001 |  | 3.50[2.60,5.00] | 4.90, 3.60 | 0.596 | 0.607 | 0.352 | 0.694 |
| Lymphocyte (×10^9^) | 1.50[1.20, 1.92] | 1.20 [0.87, 1.48] | 0.011 |  | 1.70 [1.40, 2.00] | 1.40, 1.50 | 0.315 | 0.194 | 0.264 | 0.038 |
| Monocyte (×10^9^) | 0.50[0.37, 0.60] | 0.63[0.37, 0.83] | 0.191 |  | 0.41 [0.33, 0.60] | 0.39, 0.40 | 0.833 | 0.328 | 0.352 | 0.136 |
| NLR | 2.09[1.52, 3.10] | 4.10[3.80, 5.33] | 0.000 |  | 2.17[1.40, 2.75] | 3.50, 3.00 | 0.079 | 0.647 | 0.132 | 0.117 |
| PLR | 130.59[101.43, 163.41] | 185.72[158.15, 255.83] | 0.001 |  | 113.50[85.26,158.00] | 164.29, 102.50 | 0.768 | 0.290 | 0.132 | 0.039 |
| CRP | 1.28[1.28, 2.89] | 2.65[1.44, 4.95] | 0.009 |  | 1.28[1.28, 4.24] | 1.28, 1.62 | 0.709 | 0.173 | 0.198 | 0.611 |

p_1_ value: male patients in non-rupture group vs male patients in rupture group;

p_2_ value: female patients in non-rupture group vs female patients in rupture group;

p_3_ value: male patients in non-rupture group vs female patients in non-rupture group;

p_4_ value: male patients in rupture group vs female patients in rupture group;

p_5_ value: male patients vs female patients
